# Effect of high-pressure pulsatile lavage versus manual rinsing on bone cement penetration in total knee arthroplasty: a randomized clinical trial

**DOI:** 10.1186/s43019-025-00298-x

**Published:** 2025-11-04

**Authors:** Eduard Ramírez-Bermejo, Manel Fa-Binefa, Jorge Pilco-Inga, Marcos Jordán-Sales, Xavier Aguilera-Roig, J. C. González-Rodríguez

**Affiliations:** 1 Department of Orthopaedic Surgery, Hospital de La Santa Creu I Sant Pau. C/ Sant Quintí 89, 08041 Barcelona, Catalunya Spain; 2 Universitat Autònoma de Barcelona (UAB), Barcelona, Spain; 3 Sant Pau Biomedical Research Institute (IIB Sant Pau), Barcelona, Spain

**Keywords:** Total knee arthroplasty, Total knee arthroplasty cementation, Bone cement penetration, High pressure pulsatile lavage, Pulsatile lavage

## Abstract

**Background:**

Bone irrigation is a crucial step in cemented total knee arthroplasty procedures to promote maximal cement penetration and interdigitation into the cancellous bone. However, it is not clear which type of bone irrigation achieves the best results. This study aimed to compare the efficacy of high-pressure pulsatile lavage versus manual rinsing in promoting bone cement penetration during total knee arthroplasty.

**Methods:**

We conducted a single-center, prospective, randomized, controlled clinical trial in 100 patients undergoing primary total knee arthroplasty during 1 year. All patients were randomly allocated to either the pulsed lavage group or the non-pulsed lavage group. We assessed total cement penetration depth across all zones radiologically using anteroposterior and lateral radiographic views in postoperative X-rays taken on the first day after surgery and segmenting them into ten zones according to the Knee Society Scoring System (KSSS).

**Results:**

The patient cohort included 100 individuals with an average age of 75 years (standard deviation [SD] 5.7); 73% were female. The mean total bone cement penetration values in both anteroposterior (AP) and lateral views were 10.77 mm (SD 5.95) and 4.85 mm (SD 3.33) for manual lavage, and 11.34 mm (SD 6.26) and 5.23 mm (SD 3.50) for pressurized lavage. We observed no significant differences between the two groups after adjusting for multiple variables.

**Conclusions:**

High-pressure pulsatile lavage showed no significant differences in enhancing bone cement penetration compared with manual lavage as measured by the KSSS total knee arthroplasty bone cementation scale in X-rays taken on the first postoperative day.

*Level of Evidence* Level I—Therapeutic randomized controlled trial.

*Trial registration* Clinicaltrials.gov Register-NCT06032507

## Background

Cemented total knee arthroplasty (TKA) is the gold standard for surgical treatment of knee osteoarthritis and consistently delivers excellent clinical and functional results, as documented in numerous studies [1, 2]. Survival rates of 90% over 15 years can be achieved [3]. However, a significant cause for revision is the aseptic loosening of the tibial component. [4] This loosening can occur at the bone-cement interface or at the cement-implant interface, highlighting the pivotal role that the cementing technique plays in securing initial fixation of TKA components by reducing micromotion and thereby enhancing implant stability and longevity [5, 6].

Enhancing the bone-cement interface through optimal cement penetration and interdigitation into the cancellous bone is a critical objective of bone irrigation in the cemented TKA surgical technique [7]. Several irrigation techniques, such as manual rinsing, brush, and high-pressure pulsed lavage have been performed before cementing in cadaveric studies [8–15]. However, in vivo studies analyzing the effect of the various irrigation techniques on cement penetration remain limited. Current comparative studies evaluating cement penetration have been conducted exclusively on cadaveric specimens, demonstrating greater cement penetration with the use of pulsatile lavage. However, to our knowledge, there are no comparative in vivo studies in the literature directly assessing differences in cement penetration between pulsatile and manual lavage techniques. This highlights a significant gap in the current evidence base regarding clinical outcomes associated with different lavage methods in live patients [16–18].

The purpose of the present study was to compare the effectiveness of high-pressure pulsatile lavage and manual rinsing in promoting penetration of bone cement during TKA procedures. We hypothesized that high-pressure pulsatile lavage could increase the intrusion of the cement into cancellous bone.

## Methods

### Study design

**Fig. 1:**
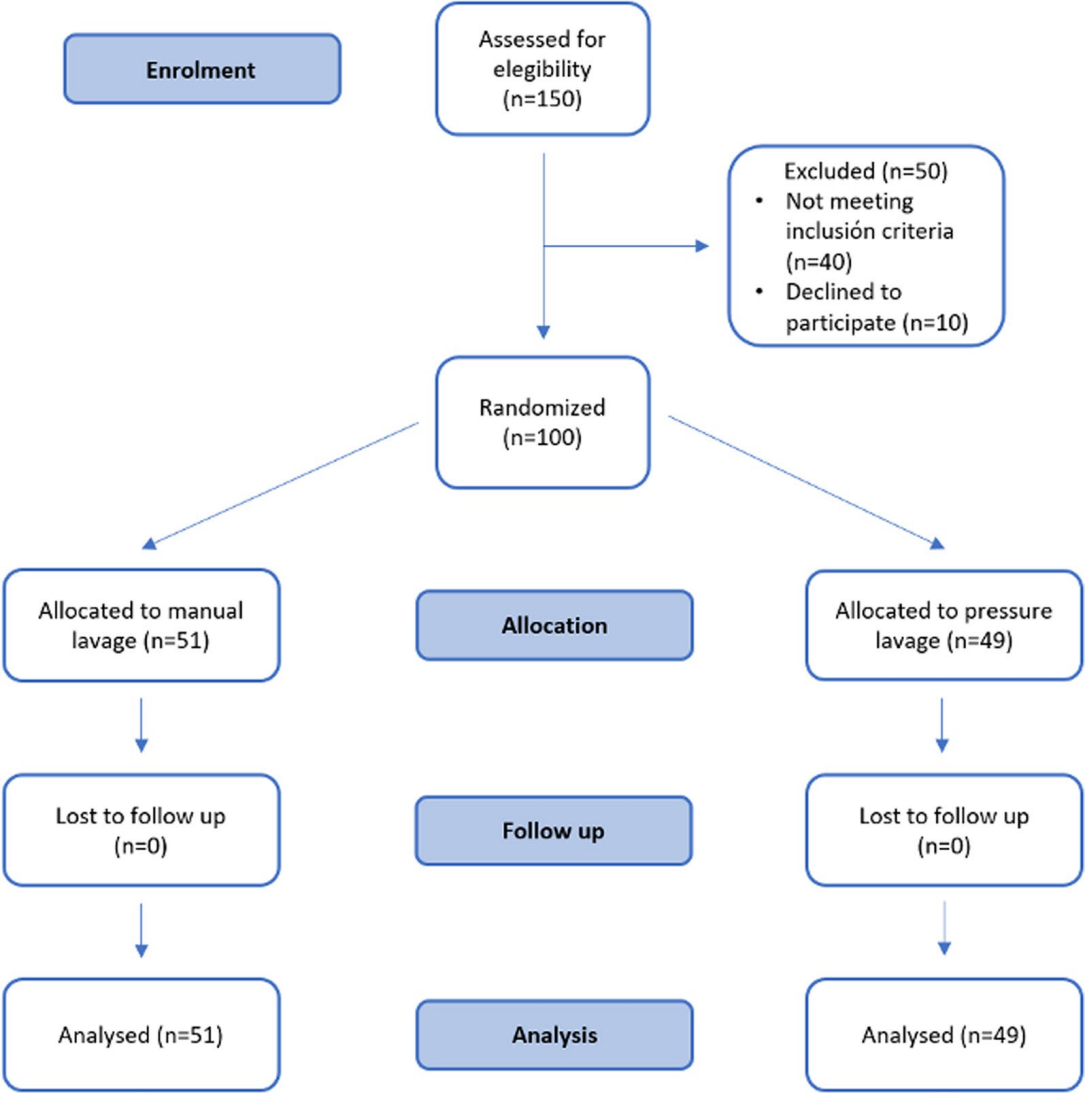
Flow diagram showing the study design

A single-center, prospective, therapeutic, randomized clinical trial was carried out from September 2021 to September 2022. Patients undergoing primary TKA were randomly assigned to either a high-pressure pulsatile lavage group or a manual rinsing group prior to TKA cementation. The flow chart diagram is shown in Fig. 1. The study was approved by the Ethics Committee and Research Institute at our institution. The study protocol was registered at ClinicalTrials.gov.

### Study population

All patients were eligible to participate if they had clinical symptoms of knee osteoarthritis but had not responded to conservative treatment measures with the established criteria and recommendations of the knee surgeon’s team [19]. Patients were excluded if they had metal allergies, active or latent knee sepsis, dysfunction of the extensor mechanism, morbid obesity, severe peripheral vascular disease, or major psychiatric or neurological disorders. All participants provided informed consent to participate in the study and consent for publication of the results.

### Randomization

Patients who met the inclusion criteria were consecutively included in the study on the basis of the inclusion criteria and upon signing the informed consent form. Once deemed eligible, they were randomized to receive either high-pressure pulsatile lavage (high-pressure group) or manual rinsing (manual group) prior to TKA cementation. The randomization model was devised in blocks of ten using a 1:1 ratio (IBM Corp. Released 2019. IBM SPSS Statistics for Windows, Version 26.0. Armonk, NY: IBM Corp). Allocation was facilitated through sequentially numbered, sealed, opaque envelopes that were opened immediately before the bone cementation process.

### Baseline data

Patient characteristics, obtained from patients’ clinical charts, were age (years), weight (kg), body mass index (BMI; range), smoking habit, previous disease (high blood pressure, diabetes mellitus, coronary heart disease, peripheric arteriopathy, rheumatoid arthritis, or chondrocalcinosis), and the radiographic grade of osteoarthritis assessed by our knee orthopedics team following the Kellgren–Lawrence classification [20].

### Intervention—Surgical technique

All TKA were implanted according to a standard protocol. Cemented TKA was performed by four senior knee surgeons following the standard medial parapatellar approach with tourniquet. A cemented Triathlon^®^ prosthesis was implanted (Stryker, Kalamazoo, Michigan, USA). For the cementation, 60 g of Simplex (Stryker, Kalamazoo, Michigan, USA) bone cement without antibiotic was applied.

### High-pressure pulsatile lavage versus manual rinsing

**Fig. 2:**
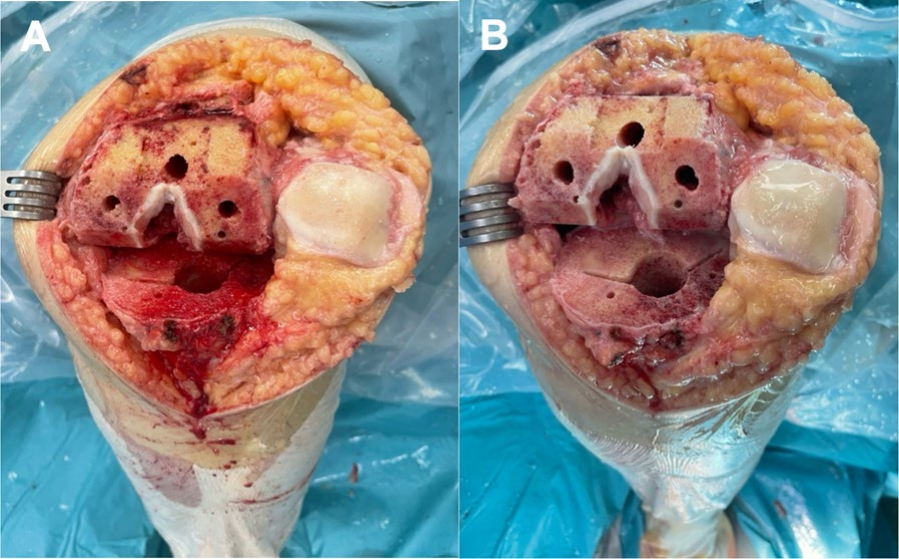
Surgical image of the knee immediately before (**A**) and after (**B**) manual-rinsing lavage

**Fig. 3:**
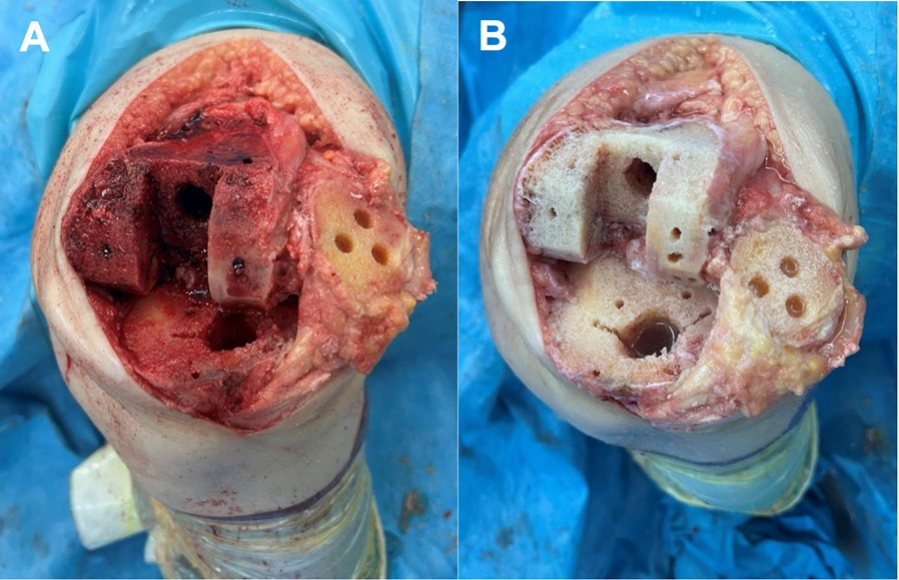
Surgical image of the knee immediately before (**A**) and after (**B**) high-pressure pulsatile lavage

Bone irrigation was performed following assignment by means of sealed randomization envelopes opened during surgery immediately before the cementation process. Both manual rinsing and high-pressure pulsatile lavage were performed for both approaches using 3 L of saline solution for 5 min. Manual rinsing was administered using a bottle with irrigation solution NaCl 0.9% sterile and endotoxin-free (B Braun, Melsungen, Hessen, Germany) (Fig. 2). High-pressure pulsatile lavage was administered using Cleanest™ (Sungo Europe B.V, Netherlands) (Fig. 3). The cement was manually applied and digitally pressurized on the tibia bone surface before implanting the tibial tray.

### Outcome: bone cement penetration

Bone cement penetration in TKA was evaluated using the standard Knee Society Scoring System (KSSS), recognized as an accessible, reliable, and reproducible method. [19]. Evaluations were conducted by three distinct observers: a senior orthopedic surgeon, a junior orthopedic surgeon, and a senior resident. None of them were involved in the patients’ treatment. All radiographs were anonymized and coded to remove patient identifiers and surgical details, and contained no information on the lavage method used. Standardized postoperative digital anteroposterior (AP) and lateral radiographs were collected and a standard magnification, followed by obliquity control to ensure the tibial plateau height was visible on both the lateral and medial aspects in the AP view, and on the anterior and posterior aspects in the lateral view.

**Fig. 4:**
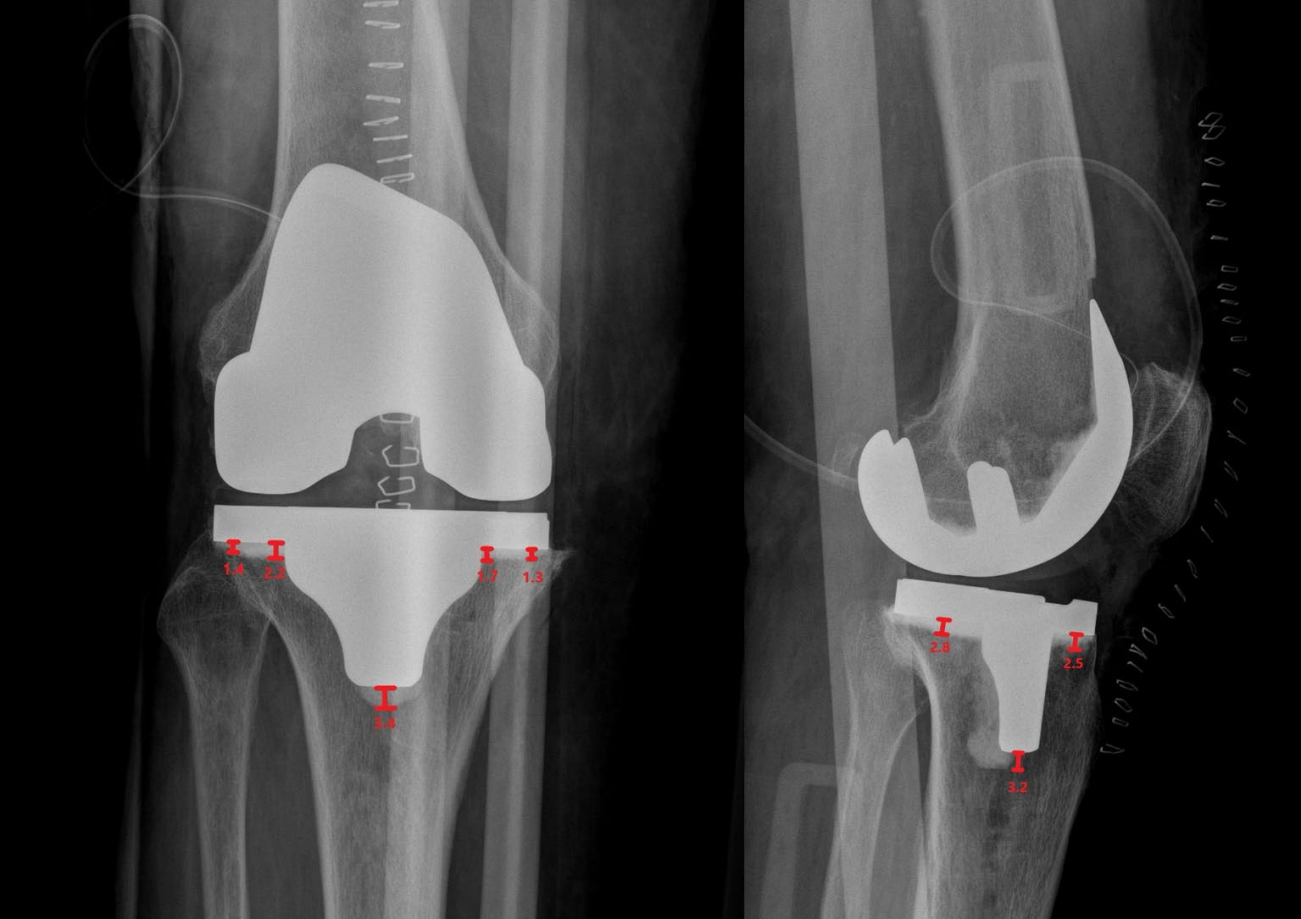
Radiographic measurement of cement penetration in both AP and lateral views in total knee arthroplasty

Radiographic assessment involved delineating a perpendicular line from the lower tibial plateau to the deepest accessible bone-to-cement transition zone within each of the ten KSSS zones (seven in the AP and three in the lateral view). [21–25]. All observations were performed using the Impax BI tool © (Version 11.1.1), by AGFA Healthcare (Fig. 4).

The cumulative cement penetration in the seven AP zones and three lateral zones was expressed as the average cement penetration for each patient.

### Data analyses

Sample size was calculated on the basis of the primary outcome, that is, the sum of cement penetration measurements in ten KSSS zones. This measurement was reported in previous literature and it was assumed that the variation would be approximately 11 mm (SD), with a minimum difference to assess between groups of 6.5 mm (average thickness of 0.65 in the ten zones) [21]. We estimated that losses would not exceed 10%. The value for a type I error was specified at 5% (*α* = 0.05, two-sided). The value for a type II error was specified at 0.2 with a minimum power of 80%. The number of patients needed to be included in this clinical trial in a perfectly balanced design was calculated as 100, randomized between the 2 groups in a ratio of 1:1 (GranMo package, V 7.12, IMIM, Barcelona, Spain). An exploratory two-sided noninferiority analysis with a 90% confidence interval was performed for the cumulative cement penetration measured on postoperative AP and lateral radiographs. The noninferiority margin was set at 2 mm on the basis of the cumulative cement penetration scale reported by Sasaki et al. [26]

For the statistical analysis between the two groups we compared the cumulative scores of bone cement penetration. We analyzed categorical variables, presented as number with percentage, using the *χ*^2^ test or Fisher’s exact test. Continuous variables, presented as mean (standard deviation, SD) or median (interquartile range, IC), were analyzed using the Student’s *t* test and Mann–Whitney *U* test, depending, respectively, on whether the distribution was normal. Secondary sensitivity analysis was performed excluding the metaphyseal zones that may be influenced by bone density and limited accessibility to pulsatile lavage (zones 5, 6, and 7 in the anteroposterior view and zone 3 in the lateral view) [25]. All results are reported with 95% confidence intervals (CI). A *p*-value < 0.05 was considered statistically significant. Statistical analysis was performed using the IBM-SPSS^®^ package V26.

## Results

### Population characteristics

The study included 100 patients. Their mean age was 75.25 years (SD 5.78) and 73% were women. All participants were enrolled in the study and underwent TKA between September 2021 and September 2022 and all completed follow-up until postoperative X-ray control. No statistical differences were found between groups for any other patient characteristics. (Table 1).

**Table 1:** Patient baseline characteristics

	Total (*N*= 100)	Manual lavage (*n* = 51)	Pressure lavage (*n* = 49)	*p*-Value
Women	73	37	36	1.000
Men	27	14	13	1.000
Age (years)	75.1 ± 6.5	75.3 ± 5.8	75.0 ± 7.3	0.860
Body mass index	29.7 ± 4.52	29.8 ± 4.1	29.6 ± 4.9	0.890
Weight (kg)	76.7 ± 14.05	76.9 ± 12.9	76.5 ± 15.2	0.880
Smoking	9	2	7	0.089
High blood pressure	62	33	29	0.681
Diabetes mellitus	19	11	8	0.613
Coronary heart disease	6	4	2	0.678
Peripheric arteriopathy	12	5	7	1.000
Rheumatoid arthritis	2	1	1	0.742
Chondrocalcinosis	23	13	10	0.358
Keller–Lawrence grade 3 Keller–Lawrence grade 4	45 55	24 27	21 28	0.693

### Bone cement penetration

**Fig. 5:**
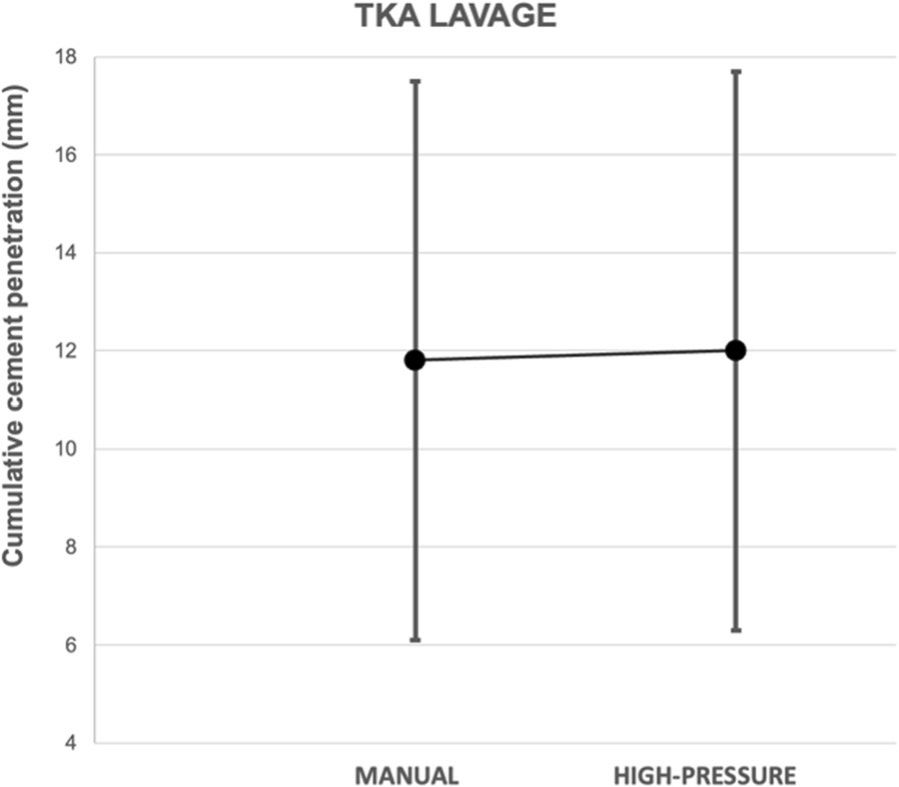
Differences between manual lavage and pressure lavage regarding cumulative cement penetration for zones 1–7 in the anteroposterior view

**Fig. 6:**
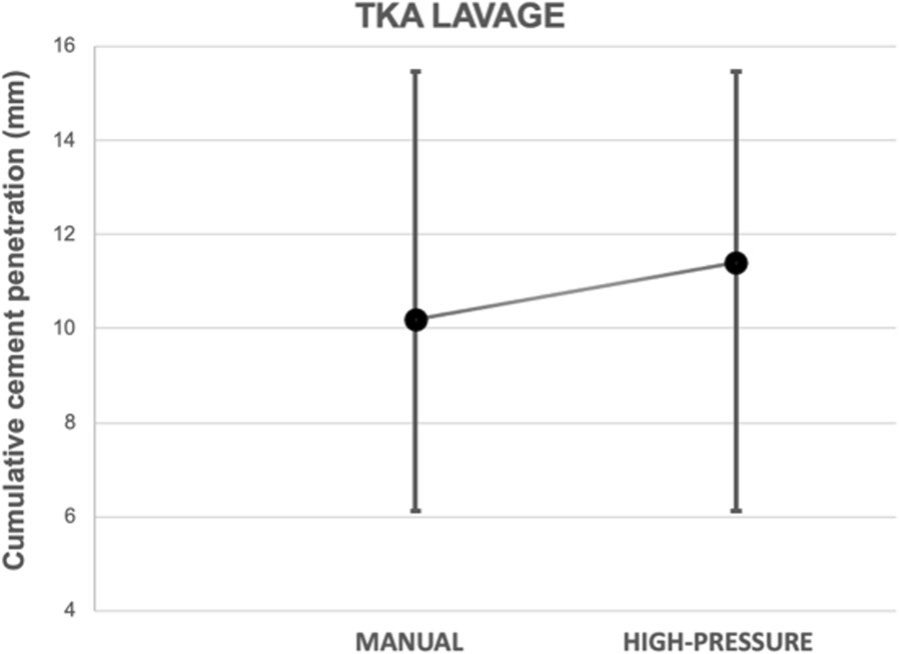
Differences between manual lavage and pressure lavage regarding cumulative cement penetration for zones 1–3 in the lateral view

The average cumulative bone cement penetration on the anteroposterior view was 11.8 mm (SD 5.7) for manual lavage and 12.0 mm (SD 5.7) for pressure lavage (n.s.) (Table 2, Fig. 5). On the lateral view, it was 10.2 mm (SD 5.5) for manual lavage and 11.4 mm (SD 5.2) for pressure lavage (Table 2, Fig. 6). These differences were not statistically significant for cumulative cement penetration in the KSSS cementation zones, either in the AP view (*p* = 0.644) or in the lateral view (*p* = 0.582) (Table 2). Exploratory noninferiority analysis showed that manual lavage was noninferior to pressure lavage on both AP and lateral cumulative views (AP 0.20 mm (CI −1.69, 2.09) and lateral view 1.20 mm (CI −0.58, 2.98), *p* = 0.001)*. The sensitivity analysis, excluding zones 5, 6, and 7 in the anteroposterior view and zone 3 in the lateral view, showed no significant differences between groups.* The mean cumulative cement penetration in the anteroposterior view (zones 1–4) was 7.1 mm (95% CI 6.2, 8.0) for manual lavage and 6.9 mm (95% CI 5.9, 7.9) for pressure lavage (*p* = 0.644), while in the lateral view (zones 1–2) it was 6.2 mm (95% CI 5.3, 7.1) and 6.8 mm (95% CI 5.9, 7.7), respectively (*p* = 0.589).

**Table 2:** Knee Society Scoring System for total knee arthroplasty

KSSS cementation zones	Total (*N* = 100)	Manual lavage (*n* = 51)	Pressure lavage (*n* = 49)	
Anteroposterior view				
1	1.5 ± 1.1	1.6 ± 1.1	1.4 ± 1.1	
2	2.2 ± 1.4	2.0 ± 1.2	2.3 ± 1.7	
3	1.8 ± 1.2	1.8 ± 1.0	1.8 ± 1.3	
4	1.5 ± 1.0	1.7 ± 1.0	1.4 ± 0.9	
5	0.4 ± 0.6	0.4 ± 0.7	0.3 ± 0.5	
6	4.0 ± 4.5	3.7 ± 4.3	4.4 ± 4.6	
7	0.5 ± 0.9	0.5 ± 0.9	0.5 ± 1.0	
Average cumulative cement penetration	11.9 ± 5.7	11.8 ± 5.7	12.0 ± 5.7	*p* = 0.644 *p* = *0.659*^*a*^
Lateral view				
1	3.4 ± 1.6	3.2 ± 1.7	3.6 ± 1.5	
2	3.1 ± 1.8	3.0 ± 1.9	3.2 ± 1.6	
3	4.3 ± 4.7	4.1 ± 4.5	4.6 ± 5.0	
Average cumulative cement penetration	10.8 ± 5.3	10.2 ± 5.5	11.4 ± 5.2	*p* = 0.582 *p* = *0.600*^*a*^

The values are given as the average ± standard deviation

^*a*^Multivariable model with no statistically significant differences were observed for the included covariates

## Discussion

The main finding in this study was that no differences were found between high-pressure pulsatile lavage and manual-rinsing lavage regarding cement penetration into cancellous bone during TKA, as assessed through the KSSS in X-rays taken on the first postoperative day. To our knowledge, the present study is the first randomized clinical trial to analyze the relation between the use of pulsed lavage and tibial bone cement penetration in primary TKA. Although the irrigation technique before cementing is of great importance to avoid early failures and to achieve high survival rates on TKA, [5, 6] few studies have evaluated cement penetration after pulsed lavage or compared this with other forms of lavage. Furthermore, most studies to date have been carried out in cadaver knee specimens.

In a comparative study in ten cadaveric specimens, Boontanapibul et al. [9] found that combining innovative pressurized carbon dioxide lavage and pulsatile normal saline irrigation before TKA implantation produced significantly deeper cement penetration into cancellous bone than pulsatile normal irrigation alone (1.90 ± 0.39 mm versus 1.21 ± 0.21 mm, *p* = 0.04). They calculated cement penetration using digital vernier calipers after the proximal tibia was cut on three planes following TKA implantation; they did not, however, include a radiographic assessment. In another study, Schlegel et al. [13] reported their results from of a series of six pairs of cadaveric tibiae, showing that cement penetration was greater in the pulsed lavage group combined with finger packing of bone cement than on syringe lavage and gun cementing (1.47 mm in the pulsed lavage group and 0.4 mm in the syringe lavage group; *p* = 0.004). They calculated cement penetration by computed tomography (CT) but applied a release agent to the tray undersurface prior to implantation, and evaluated CT after implant removal. Previously, Schlegel et al. [14] evaluated scans of bone cement penetration in six pairs of cadaveric specimens in TKA. They found that cement penetration was significantly higher in the pulsed lavage group than in the syringe lavage group (1.32 mm versus 0.79 mm; *p* = 0.031). They calculated cement penetration by CT with a median cement penetration depth and range for the entire cemented area. The present study was performed in vivo in 100 patients. We found no differences in cement penetration between high-pressure pulsatile lavage and manual-rinsing lavage (11.8 versus 12.0 mm on anteroposterior view; *p* = 0.644; and 10.2 versus 11.4 mm on lateral view; *p* = 0.582). Cement penetration was calculated by postoperative X-ray according to the KSSS. Manual lavage was noninferior to pulsatile lavage for cement penetration, with values above the threshold linked to a reduced risk of radiolucent lines > 2 mm. Higher cement penetration values observed in zone 6 of the anteroposterior view and zone 3 of the lateral view likely correspond to the metaphyseal region, where local variations in bone density and the limited accessibility of pulsatile lavage may influence cement distribution [25]. However, the sensitivity analysis excluding these areas yielded no significant differences between manual and pressure lavage groups, suggesting higher penetration values in these zones.

In addition to clinical considerations, practical factors related to surgical efficiency and resource utilization should also be taken into account. Manual rinsing, in contrast to pulsatile lavage, is associated with reduced economic cost, shorter procedural time, and decreased demand for operative equipment.

Regarding in vivo studies, Ritter et al. [18] divided 221 patients treated with TKA into three groups on the basis of the preparation of the surface of the bone and the technique of the cement application. Evaluating radiolucency at the bone-cement interface at 1 year and 3 years postoperatively they found that rates of radiolucency adjacent to the tibial components of the knees that had been prepared with irrigation of the bone surfaces with a syringe and manual packing of the bone cement were not only higher than those in knees that had been prepared with high-pressure lavage of the bone surfaces and manual packing of the cement, but also when compared with knees that had been prepared with high-pressure lavage and pressure injection of low-viscosity methylmethacrylate cement. However, they did not evaluate cement penetration, the study was not randomized, and the TKA analyzed was an all-polyethylene tibial plateau model.

In the present study, we evaluated cement penetration using the KSSS scale on AP and lateral plain X-rays. [19] The use of this scale and comparable methods [21] to assess cement penetration in plain X-rays has been recognized as a reliable method [21, 23], demonstrating up to 0.9 consistency for both interobserver and intraobserver agreement [23].

Our study has several limitations. One is related to variability in X-ray imaging as patient position may alter results. However, this bias was minimized by means of an X-ray protocol evaluating tibial plateau obliquity and assessing our results according to this. Another limitation could be the use of X-ray rather than CT; however, X-ray reduces patient radiation. Furthermore, the only scale available to date to evaluate bone cementation is the KSSS TKA bone cementation scale, and the number of published studies directly addressing cement penetration using this method is limited, which may restrict comparison with previous literature. The performance of all TKAs by a limited group of four surgeons may also be considered a potential limitation regarding external validity. Finally, our sample size was not based on previous studies in vivo following the same system, and a larger sample size may find statistically significant differences.

## Conclusions

High-pressure pulsatile lavage did not demonstrate significant differences in enhancing bone cement penetration compared with manual lavage, as measured by the KSSS TKA bone cementation scale in X-rays taken on the first postoperative day.

## Data Availability

The datasets generated and analyzed during the current study are not publicly available due to restrictions imposed by the hospital ethics committee regarding patient data confidentiality.

## Abbreviations

TKA: Total knee arthroplasty
KSSS: Knee society scoring system
AP: Anteroposterior
